# Simulation of Oscillopsia in Virtual Reality

**DOI:** 10.22599/bioj.112

**Published:** 2018-06-19

**Authors:** David Randall, Helen Griffiths, Gemma Arblaster, Anne Bjerre, John Fenner

**Affiliations:** 1 University of Sheffield, GB

**Keywords:** Oscillopsia, Virtual Reality, Nystagmus, Simulation, Ocular Motility

## Abstract

**Purpose:**

Nystagmus is characterised by involuntary eye movement. A proportion of those with nystagmus experience the world constantly in motion as their eyes move: a symptom known as oscillopsia. Individuals with oscillopsia can be incapacitated and often feel neglected due to limited treatment options. Effective communication of the condition is challenging and no tools to aid communication exist. This paper describes a virtual reality (VR) application that recreates the effects of oscillopsia, enabling others to appreciate the condition.

**Methods:**

Eye tracking data was incorporated into a VR oscillopsia simulator and released as a smartphone app – “Nystagmus Oscillopsia Sim VR”. When a smartphone is used in conjunction with a Google Cardboard headset, it presents an erratic image consistent with oscillopsia. The oscillopsia simulation was appraised by six participants for its representativeness. These individuals have nystagmus and had previously experienced oscillopsia but were not currently symptomatic; they were therefore uniquely placed to judge the app. The participants filled in a questionnaire to record impressions and the usefulness of the app.

**Results:**

The published app has been downloaded ~3700 times (28/02/2018) and received positive feedback from the nystagmus community. The validation study questionnaire scored the accuracy of the simulation an average of 7.8/10 while its ability to aid communication received 9.2/10.

**Conclusion:**

The evidence indicates that the simulation can effectively recreate the sensation of oscillopsia and facilitate effective communication of the symptoms associated with the condition. This has implications for communication of other visual conditions.

## Introduction

Nystagmus is an eye movement disorder in which the individual experiences repetitive involuntary eye motion. It has been estimated to affect 0.24% of the population in the UK (Sarvananthan et al. 2009). Nystagmus is broadly classified into two groups: infantile (present at birth or within the first six-months of life) and acquired (developed later in life). Those with infantile nystagmus usually adapt to the erratic eye motion during early neural development; nevertheless, it adversely affects quality of vision (e.g. visual acuity and ‘time to see’) (Dell’Osso and Jacobs 2002; Erichsen et al. 2010). Acquired nystagmus patients rarely adapt and perceive the world as constantly in motion – a debilitating symptom known as oscillopsia. A substantial number of infantile nystagmus patients also experience infrequent oscillopsia, although exact numbers are not known (Abadi et al. 1999).

The nystagmus waveform can present as one of two main characteristic types: Pendular (repetitive slow eye motion of equal speed in either direction) and jerk (repetitive eye motion with a fast phase in one direction and slow phase in the other direction). The direction of eye movement can be horizontal, vertical, rotational or a combination of these. Regardless of the type of motion and its severity, oscillopsia is often incapacitating, typically resulting in nausea, vertigo and loss of balance; disabling effective interaction with the world around them. Patients with oscillopsia currently have few opportunities for relief: there are no reliable treatment options and as a result, they struggle to live independent lives (Tilikete and Vighetto 2011; Anson et al. 2018; Rowe et al. 2018; Tse et al. 2017). Anecdotally, a frequent complaint among those with oscillopsia is that they feel neglected and struggle to describe the condition and explain the effect it has on their everyday lives. Tools to help communicate the condition would be of benefit but no authentic recreation of oscillopsia currently exists for patient use. This paper describes the application of virtual reality (VR) technology to simulate the condition to aid communication.

The VR simulation has been released as a smartphone app called “Nystagmus Oscillopsia Sim VR”, for both Android and iPhone app stores, to be viewed using a Google Cardboard headset. The app was produced with the following specific aims in mind:

1. To aid description of the condition.
2. To raise awareness of nystagmus among the general population.
3. To be an educational tool for clinicians and other healthcare professionals learning about nystagmus and oscillopsia.

The purpose of this paper is to communicate the production and utility of the app.

## App production

Commercially available, clinical grade eye tracking hardware (Eyelink 1000 Plus) (Research 2017) was used to record eye movements in three individuals, each with a different type of nystagmus (horizontal jerk, vertical jerk and horizontal pendular). First, a calibration procedure was performed on each participant. They were then asked to focus on a stationary cross so that the recorded eye movements corresponded to the involuntary movements resulting from the individual’s nystagmus in primary position. Angular rotation measurements (in the horizontal or vertical direction) were imposed onto the ‘virtual eyes’ in the Unity game engine via a script written in C# (a programming language native to Unity).

**Figure 1:**
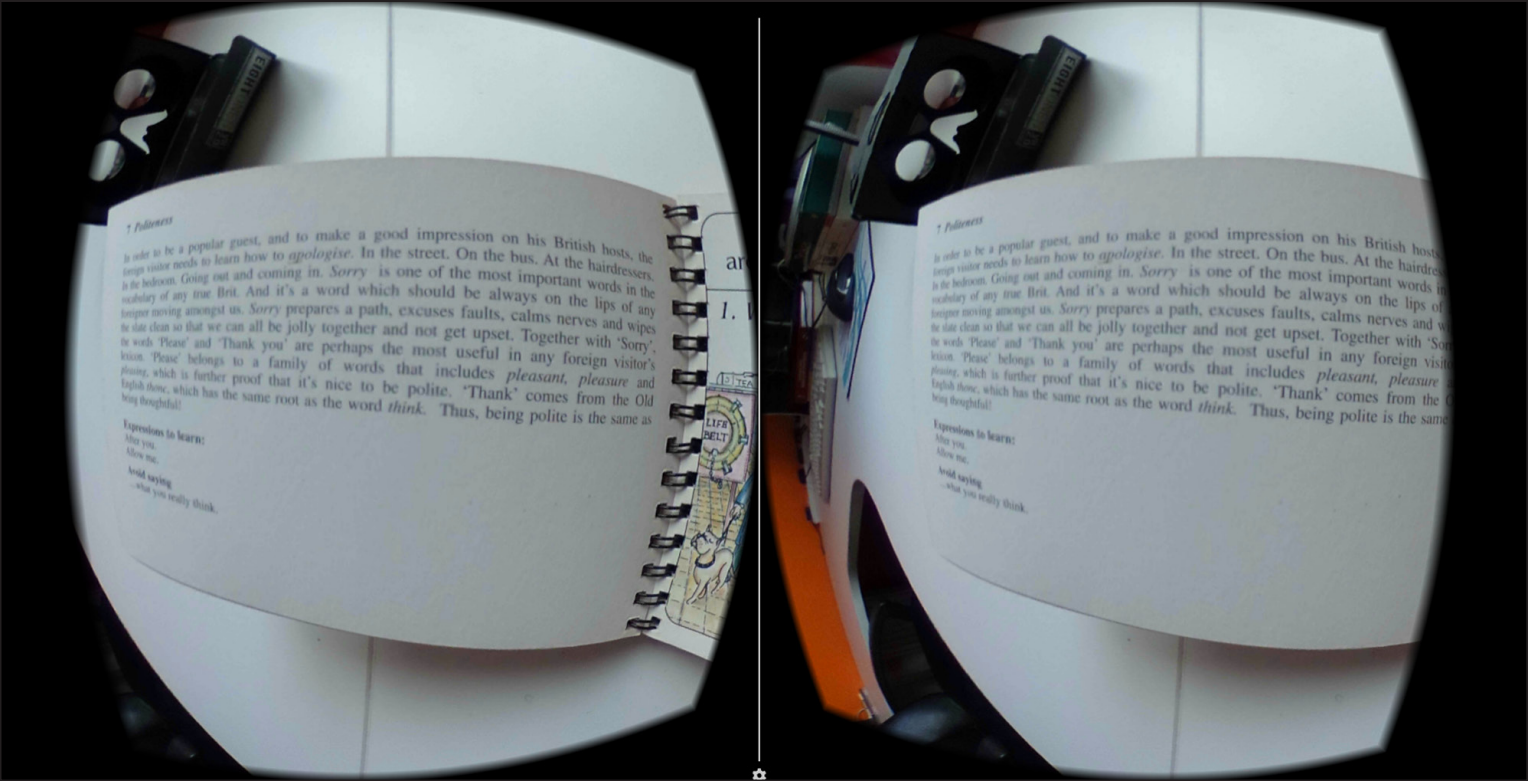
Reading Scene showing a stereo view of a large-print book to demonstrate an example of the difficulties encountered by those with oscillopsia in everyday life.

Four different environments were produced in which to experience oscillopsia. The scenes consist of real-life recordings/images, rather than artificial computer-generated graphics, to more closely reflect real life. Each was designed to loosely align with some of the difficulties encountered by those with the condition and consisted of: a traffic scene, book reading scene, ballet scene and an optometrist’s room. The different eye movement recordings and the different scenes were accessible through a ‘main menu’ scene incorporating virtual buttons accessed by gaze selection. Figure 1 shows a screen capture of the ‘reading Scene’ with the user positioned in front of a large print book.

## Patient Feedback

### Feedback Acquisition

Six patients with infantile nystagmus who had previously experienced oscillopsia were identified from an existing group of University of Sheffield clinical examination volunteers (ethical approval granted via a local ethics committee – Application No. 009207). Participants had their eye movements recorded and transferred into the virtual reality app. The personalised simulation and ‘typical’ oscillopsia simulation in the published app were then viewed by the participant and they were asked to complete a questionnaire. The questionnaire first acquired information regarding the individual’s condition, how regularly oscillopsia was experienced and approximate length of time since their last episode. The remainder of the questionnaire contained four questions to ascertain whether the simulation was representative:

1. Based on your previous experience, how close does this personalised oscillopsia simulation replicate what you remember seeing?
  Scale of 1–10 (10 being perfectly replicated)
2. To what extent would you say this personalised simulation would help you describe what it’s like to have oscillopsia?
  Scale of 1–10 (10 being extremely helpful)
3. Would you consider this oscillopsia demonstration to be of use to show others for them to understand what oscillopsia is and what it is like to have it?
  Scale of 1–10 (10 being extremely helpful)
4. To what extent does the ‘typical’ (i.e. not personalised) simulation of oscillopsia help you describe what it is like to have oscillopsia?
  Scale of 1–10 (10 being extremely helpful)

A comments section was also provided on the questionnaire. The participants were given as much time as they wished to experience the oscillopsia and complete the questionnaire.

### Feedback results

**Figure 2:**
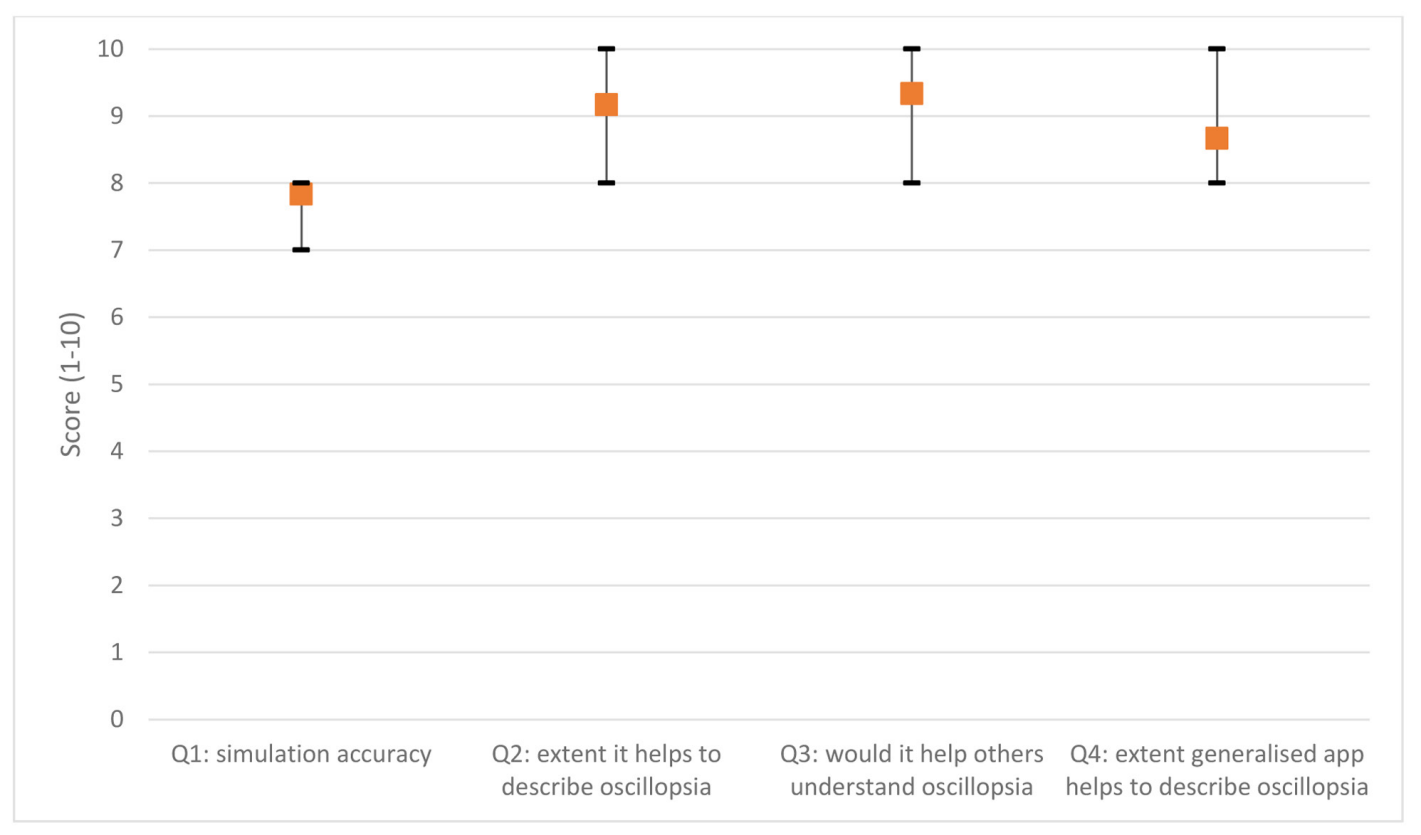
Questionnaire responses regarding the accuracy and usefulness of the virtual reality oscillopsia simulation (n = 6).

All participants had experienced oscillopsia on at least 10 occasions. Five out of six participants noted their last episode of oscillopsia to be within a week of being shown the simulation with the other participant noting their last episode to be 2–12 months earlier. Five participants had horizontal jerk nystagmus and one had horizontal pendular nystagmus. Figure 2 shows the mean results for each of the four questions, the range of values across all participants are indicated by the error bars.

## Discussion

This work sought to produce a credible simulation of oscillopsia to aid communication and raise awareness of the condition. The app was accepted for publication on Android and iPhone stores on 23^rd^ November and 1^st^ December 2016 respectively. The app has received 570 downloads on Android devices and 3115 downloads on the iPhone (28/02/2018), over a period of approximately 15 months following publication.

### App utility

One of the aims of this work was to raise awareness of the condition. The app has been downloaded 3685 times (February 2018) and this continues to rise, reaching people who might not have otherwise been aware of the debilitating symptoms of oscillopsia. However, the app (as with all VR smartphone apps) can only be run on high-end smartphones, meaning it is not available to all. As of September 2017, only 2192 of the ~14114 different Android smartphones on the market supported the oscillopsia simulation app (Google, 2017). VR compatible iPhone devices include the iPhone 5 and newer versions.

The published app incorporates three different characteristic types of nystagmus: horizontal jerk, vertical jerk and horizontal pendular. These types of nystagmus were chosen as they are thought to have the largest prevalence. Offering a simulation for three different types of nystagmus makes the app relevant to a large number of persons with nystagmus. It also educates those previously unaware to the fact that there are different types.

Beyond raising awareness and aiding communication generally, the app has found a variety of specific uses known to the authors:

- To show parents and teachers of children with nystagmus and intermittent oscillopsia (used by at least two vision support services for teachers in the UK).
- Employees to educate their employers.
- Clinicians to show a possible complication of an intervention.
- Patients to show healthcare professionals (a specific example was a multiple sclerosis patient with oscillopsia who was undergoing physiotherapy and wished to communicate the severity of his oscillopsia).

### Representativeness of the simulation

Each of the waveforms in the published app were selected by an expert in eye motility as being ‘typical’ examples of each of the nystagmus types. However, a wide variability of nystagmus waveforms exist and it is recognised that offering a single ‘typical’ waveform for each type may not create a representative experience for all individuals. Greater conformity for the individual’s nystagmus could be achieved by offering the user the option to alter the frequency and amplitude of the nystagmus waveform but this was seen as peripheral to the core goals of the app. The current ‘typical’ nystagmus simulation arguably provides an experience which enhances communication and discussion of the condition and any potential benefits of user optimisation are likely to be marginal. The usefulness of the ‘typical’ nystagmus waveforms is supported by only a small decrease in score between Question 2 (9.2/10) and Question 4 (8.7/10) in the feedback questionnaire.

The questionnaire results indicate the app is a reasonable representation of the condition with an average score of 7.8/10 (Question 1) and would help others understand, with an average score of 9.3/10 (Question 3). The usefulness of the app to help describe the condition received encouragingly high scores (9.2 and 8.7 for the personalised and typical simulations respectively). The fact that a comparable score was given to the typical simulation on the published app (relative to the personalised simulation), shows its utility to the general nystagmus population. However, a larger cohort may include participants for which the typical nystagmus waveforms are less representative and limit their capability to aid communication. Despite subjectivity in using the questionnaire, responses were so positive there can be little doubt in the utility of the VR simulation and has confirmed the app’s positive role in aiding communication of the condition.

### Limitations

Several simplifications were introduced when replicating the waveforms in VR that produce minor inaccuracies in the oscillopsia experience, including: using data from only one eye, only using one component (horizontal or vertical) and subsampling the waveform from 500 Hz to 50 Hz. However, given the large variability in nystagmus between individuals, the perturbations and inaccuracies introduced by such simplifications are unlikely to greatly affect the experience, as reflected in the feedback of the personalised oscillopsia simulations. An important characteristic of nystagmus, not currently simulated, is the change in waveform with eye orientation. This would create a more realistic experience, but would require real-time eye tracking in VR, which is not currently available in smartphone-based VR. The current implementation of the app is adequate to have fulfilled its purpose.

People with nystagmus may have a different perception of the simulation to people without the condition (e.g. greater acceptance of retinal slip) adding a source of uncertainty regarding the results of the questionnaire. The ideal would be to find a patient cohort who had made a complete, recent recovery from acquired nystagmus; however, these individuals are rare and is an impractical consideration. The method adopted was a reasonable compromise.

### Future work

The mechanism to incorporate an individual’s personalised eye recordings into the app takes approximately 10 minutes. Being able to provide such a service provides an opportunity to offer nystagmus patients their own personalised app to be taken away on their own smartphone devices.

The incorporation of nystagmus eye movements into VR also lays the foundations for correction of nystagmus in VR. By incorporating real-time eye tracking into a VR headset, it is conceivable that the headset display could be corrected to present the oscillopsia sufferer with a stable image, potentially allowing them relief and facilitating interaction with the world around them.

There are also wider implications beyond oscillopsia. VR provides a unique environment for communicating the severity of eye conditions (e.g. tunnel vision, macular degeneration etc.) and this could conceivably be extended to diagnostics/therapeutic aims. A cursory review of the literature indicates growing interest in such areas (Daga et al. 2017; Eastgate et al. 2006; Waddingham et al. 2011; Blaha and Gupta 2014).

## Conclusion

People with oscillopsia can find it difficult to describe their condition and communicate its effects on everyday life. This paper describes a VR simulation of oscillopsia designed to raise awareness and aid communication for those affected by the condition. An app was released, free for download on Android and iPhone. The results of a questionnaire, filled in by individuals with infantile nystagmus who have experienced oscillopsia, provides confidence that the app gives a representative simulation of oscillopsia and aided communication.

## Additional File

The additional file for this article can be found as follows:

10.22599/bioj.112.s1 Patient feedback on VR oscillopsia simulation results. Contains responses to all questions in the questionnaire including the data used to generate Figure 2.
